# Insight on the Swelling Pressure–Suction Relationship of Compacted Bentonite during Hydration

**DOI:** 10.3390/ma16155403

**Published:** 2023-08-01

**Authors:** Yang Wang, Jun Teng, Qi Huang, Wei Wang, Yong Zhong

**Affiliations:** 1 School of Civil and Environmental Engineering, Harbin Institute of Technology (Shenzhen), Shenzhen 518055, China; 2 Engineering Management Center, Bureau of Public Works of Shenzhen Municipality, Shenzhen 518031, China

**Keywords:** compacted bentonite, swelling pressure–suction curve, CSS curve, stress path

## Abstract

Investigation of the swelling pressure of buffer/backfill materials is a critical aspect in the design of high-level radioactive waste (HLW) disposal repositories. In this study, to clarify the swelling pressure–suction relation for compacted bentonite upon the hydration path, constant-volume swelling pressure tests with suction control were conducted. The swelling pressure–suction curves indicated that the swelling pressure of the specimens increased significantly with increasing dry density, while the shape of the curves during hydration depended on the dry density. Moreover, the swelling pressure–suction curves exhibited a distinction between unsaturated and saturated segments divided by the critical saturated state (CSS) curve, which proves the unique existence of a CSS curve in the stress space independent of the stress path. With the introduction of the CSS curve into the s–p space, the conventional stress space of unsaturated soil could expand to that of unsaturated expansive soil. The results obtained in this study could provide the mechanical parameters for the construction of disposal repositories. In addition, the stress space with CSS curve proposed in this study provides a new approach to building constitutive models of bentonite materials.

## 1. Introduction

The development of the nuclear industry has resulted in a large amount of nuclear waste. The safe disposal of this nuclear waste, especially high-level radioactive waste (HLW), has become an increasingly urgent environmental issue. Deep geological disposal is widely accepted for the safe disposal of HLW. In this conceptual design, HLW repositories are constructed in a stable geological formation located 500–1000 m below the ground surface. A multiple barrier system consisting of both natural barriers (the geological formation) and artificial barriers (buffer/backfill materials, waste containers, and solidified waste) are utilized to prevent the leakage and migration of radionuclides, ensuring permanent isolation from the human living environment. Among these barriers, buffer/backfill materials play a crucial role in maintaining the stability of the disposal facility structure, preventing groundwater infiltration, blocking the migration of radionuclides, and limiting the diffusion of radiation and heat.

Compacted bentonite has emerged as a preferred choice for buffer/backfill materials in the disposal of high-level radioactive waste in numerous countries thanks to its low hydraulic conductivity, microporous structure, good sorption properties, and swelling capacity [1,2,3,4,5,6,7]. MX-80 bentonite extracted from Wyoming, USA, has been used in many disposal concepts in Sweden, Finland, Germany, and France. FEBEX bentonite has been extracted from the Cortiji de Archidona deposit in Spain and has been chosen by the Spanish Agency for Radioactive Waste Management as a suitable material for backfilling and sealing HLW repositories. Additionally, Kunigel V1 and FoCa bentonite have been proposed as potential buffer materials in Japan and France. In China, based on the comprehensive comparison of factors such as the location of the deposit, transportation conditions, deposit reserves, and mining technology, the Gaomiaozi (GMZ) bentonite deposit in Inner Mongolia has been determined as the preferred deposit for buffer/backfill materials used in the geological disposal in China [3,4]. 

The exceptional swelling capacity and self-healing properties of compacted bentonite make it an ideal candidate to expand and fill voids and fractures in buffer/backfill materials and surrounding rocks within the repository. This characteristic enables the formation of robust barriers, effectively safeguarding against ingress of groundwater from the surrounding geological formations and potential release of radioactive waste from the canister [8]. Therefore, investigation of the swelling behavior of compacted bentonite upon hydration assumes significant importance in the design of geological repositories.

The determination of swelling pressure upon hydration is an important aspect of the research on the swelling behavior of compacted bentonite in geological repositories. Research suggests that the swelling pressure is influenced by factors such as the mineral composition, structure, and initial dry density of bentonite. The swelling pressure of bentonite can be measured through four experimental methods: the constant volume method, free swelling–compression method, constrained swelling–compression method, and zero swelling method [9,10]. Among these methods, the constant volume method is widely used in swelling pressure tests of bentonite due to its relatively simple experimental equipment and procedures. The constant volume method is commonly employed to conduct swelling pressure tests on compacted bentonite in laboratory settings, where the specimens are saturated through water flooding [11,12,13]. Research has consistently demonstrated that the final swelling pressure of bentonite is closely related to its dry density. Wang et al. [10] found that, for the same type and mineral composition of bentonite, the ultimate swelling pressure exhibits an exponential increase as the dry density increases.

Moreover, suction-controlled swelling pressure tests have been conducted on compacted bentonite to analyze the variation in the swelling pressure under suction. Lloret et al. [14] and Yigzaw et al. [15] observed a “double-peak” shape on the swelling pressure–suction curve. First, a significant reduction in suction leads to progressive swelling pressure development up to the first peak. Then, the swelling pressure is reduced due to collapse of the soil skeleton. Finally, the swelling pressure shows an upward trend again. However, different phenomena have been reported as well. Romero et al. (2003) [16] only reported the first two zones of the curve, while Wang et al. [10] and Agus et al. [17] observed a continuous increase in swelling pressure during the suction reduction process. As there have been relatively few swelling pressure tests performed with suction control, the swelling pressure–suction relationship is not yet fully understood.

In this work, constant volume swelling pressure tests with suction control were carried out on compacted GMZ01 bentonite specimens. The swelling pressure–suction relationship of specimens with different dry densities during hydration are analyzed. Then, the existence of a critical saturated state in the swelling pressure tests is discussed with respect to the stress path. Finally, the mechanisms of different hydration paths are analyzed in the new suction–stress space with the CSS curve for expansive soil.

## 2. Materials and Methods

### 2.1. Material

The GMZ01 bentonite used in this study was sourced from Inner Mongolia, China, which has been recognized as the first choice for use as buffer/backfill material for construction of Chinese deep geological repositories [4]. The deposit contains a total of 160 million tons of bentonite, with 120 million tons of Na-bentonite reserves. The mining area covers approximately 72 km^2^. The deposit was formed during the later Jurassic period. The bentonite is characterized by its bedded structure, soapy texture, and waxy appearance. The mineralization process involved the interaction of initially formed continental volcanic sediments with groundwater and weathering [18]. The basic properties of the bentonite are summarized in Table 1 [3]. It is composed of more than 75% montmorillonite with a cation exchange capacity (CEC) of 77.3 meq/100 g (43.36% Na^+^, 29.14% Ca^2+^, 12.33% Mg^2+^, 2.51% K^+^). The liquid limit and plastic limit are 276% and 37%, respectively, and the specific gravity is 2.66.

In the specimen preparation process, the bentonite powder was initially subjected to the vapor equilibrium technique, allowing it to reach a suction of 113 MPa, which corresponds to a water content of 10.2%. Subsequently, a specific amount of powder was poured into a compaction cell with an internal diameter of 50 mm. The powder was then compacted statically to achieve the desired approximate dry density of 1.30–1.70 g/cm^3^.

### 2.2. Test Apparatus

**Figure 1:**
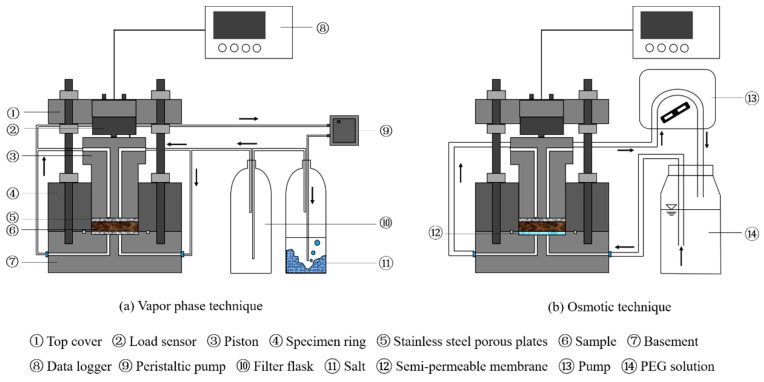
Test apparatus for the suction control swelling pressure tests.

Figure 1 illustrates the test setup utilized for conducting suction-controlled swelling pressure tests in this study. It included a constant-volume cell and suction control systems. The constant-volume cell consisted of a basement with a porous plate and a drainage system for water circulation, a specimen ring (50 mm in internal diameter) to prevent radial swelling, a stainless piston with two outlets for air expelling, and a load sensor fixed on the top for monitoring the swelling pressure during hydration. In the suction control systems, the vapor phase method [19,20], the osmotic technique [19], and the water circulation technique (Figure 1b) were employed for controlling a large suction range. To apply the vapor phase technique, the vapour of a saturated salt solution is circulated in a closed system and accelerated by a pump (Figure 1a). The relationship between the saturated salt solution and its corresponding suction proposed by Tang and Cui [20] was employed in this work. To perform the osmotic technique, a semipermeable membrane is placed under the specimen and a designed PEG (polyethylene glycol) solution is circulated underneath the semipermeable membrane (Figure 1b). The relationship between concentration of the PEG solution and its corresponding suction proposed by Delage et al. [19] was adopted in this study.

### 2.3. Test Procedures

First, a sample with a suction of 113 MPa was installed in the constant-volume cell. An initial pressure of 0.05 MPa was set to ensure good contact between the specimen and the load sensor. Subsequently, target suctions were systematically applied along the path as follows: 113–38–9–4.2–1–0 MPa. For suctions of 38 MPa, 9 MPa, and 4.2 MPa, the vapor of saturated NaCl, KNO_3_, or K_2_SO_4_ solution was circulated in the specimen utilizing the vapor equilibrium technique as illustrated in Figure 1a. For suction of 1 MPa, the vapor equilibrium technique was replaced by the osmotic technique, with PEG (polyethylene glycol) solution in a corresponding concentration circulated underneath the semi-permeable membrane (Figure 1b). Finally, to achieve the target suction of 0 MPa, the specimen was thoroughly infiltrated with distilled water.

The swelling pressure generated during the test process was continuously monitored and recorded by an automated data logger. The next suction level was applied after the swelling pressure under the current controlled suction reached a stable state.

Five tests were performed in total (Table 2) on samples with different dry densities. 

All of the tests were performed at an ambient temperature of 20 ± 0.5 °C.

## 3. Results

**Figure 2:**
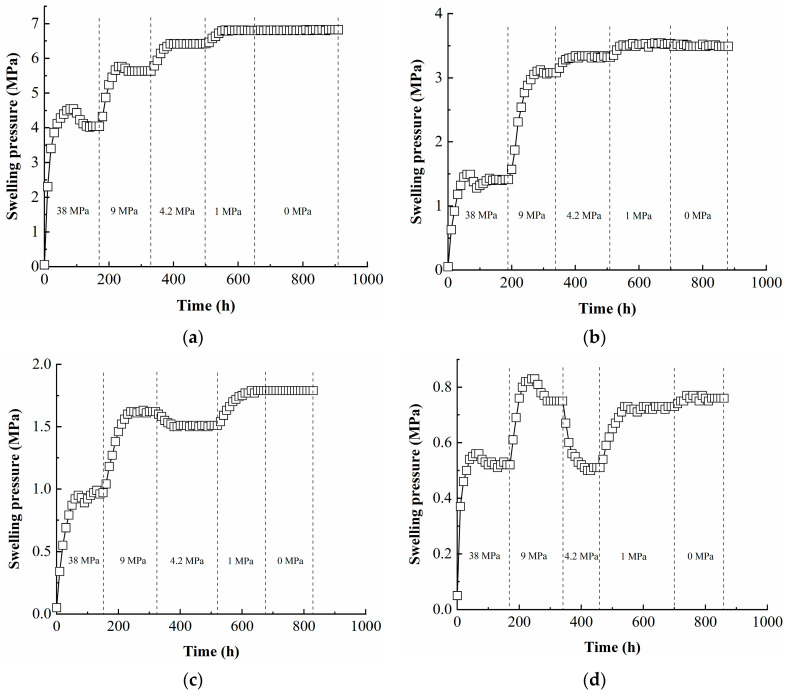

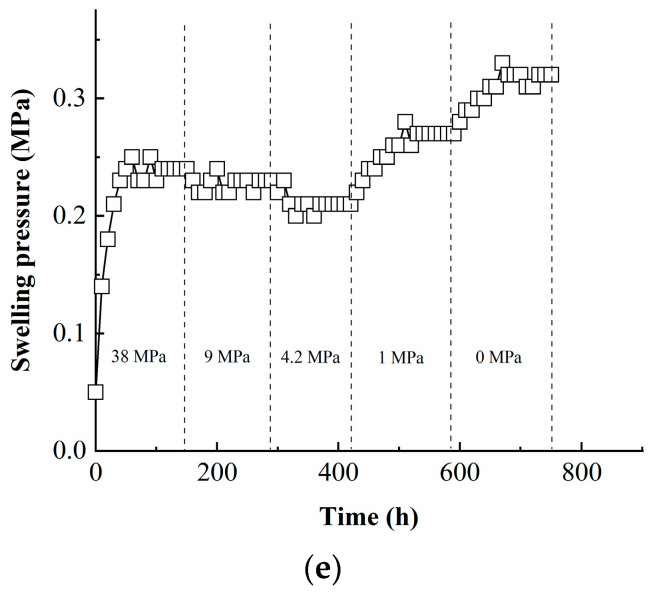
Variations in swelling pressure over time for specimens with different dry densities: (**a**) 1.70 g/cm^3^, (**b**) 1.60 g/cm^3^, (**c**) 1.50 g/cm^3^, (**d**) 1.40 g/cm^3^, (**e**) 1.30 g/cm^3^.

The variations in swelling pressure over time for samples with different dry densities are plotted in Figure 2. In the case of the specimen with a dry density of 1.70 g/cm^3^ (Figure 2a), when suction was applied up to 38 MPa the swelling pressure first increased, then decreased to 4.04 MPa. When suction was applied up to 9 MPa, the swelling pressure increased to 5.63 MPa. When suction was further applied to 4.2 and 1 MPa, the swelling pressure slightly increased to 6.42 and 6.80 MPa, respectively. When suction was finally applied to 0 MPa, the final swelling pressure was 6.82 MPa. In the case of the specimen with a dry density of 1.60 g/cm^3^ (Figure 2b), when suction was applied up to 38 MPa, the swelling pressure first increased and then decreased to 1.41 MPa. When suction was applied up to 9 MPa, the swelling pressure increased to 3.08 MPa. When suction was further applied to 4.2 and 1 MPa, the swelling pressure slightly increased to 3.32 and 3.53 MPa, respectively. When suction was finally applied to 0 MPa, the final swelling pressure was 3.49 MPa. For the specimen with a dry density of 1.50 g/cm^3^ (Figure 2c), when suction was applied up to 38 MPa, the swelling pressure first increased and then decreased to 0.97 MPa. When suction was applied up to 9 MPa, the swelling pressure increased to 1.62 MPa. When suction was further applied up to 4.2 MPa, the swelling pressure was slightly reduced to 1.51 MPa. When suction was finally applied to 1 and 0 MPa, the swelling pressure slightly increased and the final value was 1.79 MPa. In the case of the specimen with a dry density of 1.40 g/cm^3^ (Figure 2d), when suctions of 38 and 9 MPa were applied, the swelling pressure increased to 0.52 and 0.75 MPa, respectively. When suction was applied up to 4.2 MPa, the swelling pressure decreased to 0.51 MPa. As suction was further reduced to 1 and 0 MPa, the swelling pressure showed a slightly increase to 0.73 and 0.76 MPa, respectively. In the case of the specimen with a dry density of 1.30 g/cm^3^ (Figure 2e), the swelling pressure exhibited relatively lower values during the suction reduction process. When suctions of 38, 9, 4.2, 1, and 0 MPa were successively applied, the respective swelling pressures were 0.24, 0.23, 0.21, 0.27, and 0.32 MPa.

**Figure 3:**
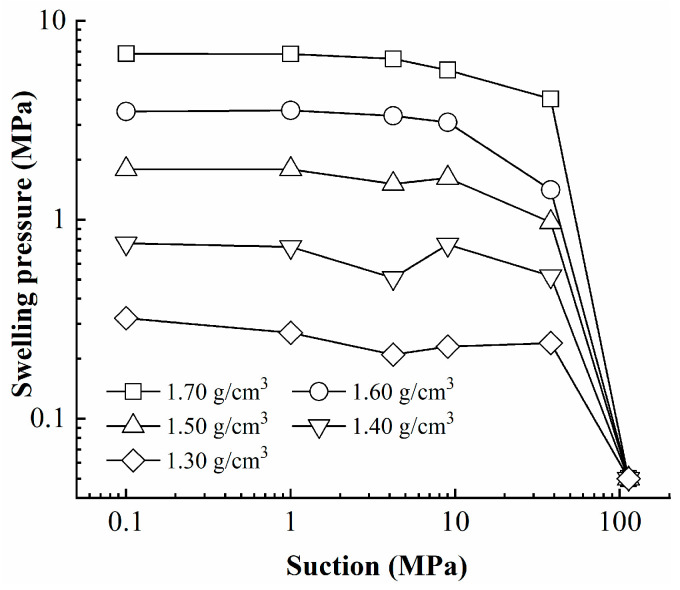
Evolution of swelling pressure with suction.

The evolution of the swelling pressure with suction are plotted for specimens in Figure 3. It can be observed that the curve of the specimen with the higher dry density is always located at a higher point, which indicates that, for the same control suction, the swelling pressure of specimens increases significantly with increasing dry density. When the dry density increases from 1.30 to 1.70 g/cm^3^, the final value of the swelling pressure after hydration increases from 0.32 to 6.82 MPa. When the dry density is lower, there are more large pores between the aggregates and the hydration expansion of the aggregates continuously fills the large pores, resulting in a smaller increase in the swelling pressure. As the dry density increases, the number of large pores between the aggregates gradually decreases, and the swelling space of the aggregate is very limited, resulting in a larger increase in the swelling pressure.

**Figure 4:**
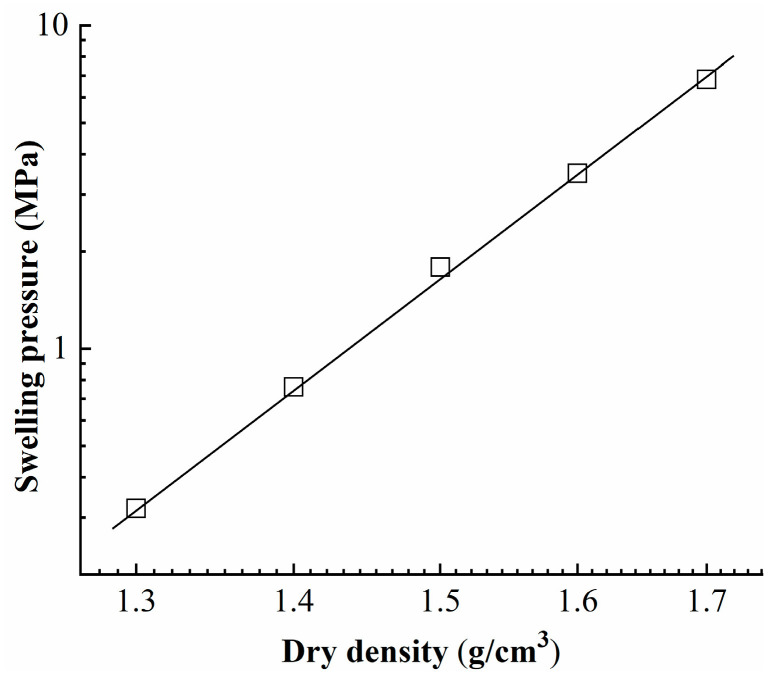
The relationship between the final swelling pressure and dry density of the specimen.

The correlation between the final swelling pressure and dry density of the specimens is potted in Figure 4. Consistent with findings from previous investigations of various bentonites [8,10,21,22], a linear relationship is observed when the data are plotted on a semilogarithmic scale.

In addition, it can be observed in Figure 3 that the shape of curve is dependent on the dry density of the specimen. For specimens with low dry densities, the swelling pressure evolution curve exhibits the characteristic that the swelling pressure initially increases, then decreases, and then increases again, which has been reported by previous researchers [14,15]. This phenomenon can be attributed to the interplay between the swelling of aggregates and the collapse of the soil skeleton. When the dry density is higher, the phenomenon becomes non-obvious, with no significant reduction process in swelling pressure.

## 4. Discussion

It can be observed from Figure 3 that the swelling pressure does not vary continuously with the decrease in suction. In the high suction zone the swelling pressure exhibits a noticeable variation with suction, whereas in the low suction zone the variation of the swelling pressure with the suction is relatively small. There seems to exist a threshold with regard to suction. When the suction reaches this threshold value, the swelling pressure no longer changes with suction. The phenomenon is especially obvious for samples with higher dry densities, such as 1.70 g/cm^3^. When the suction reaches 4.2 MPa, the swelling pressure reaches a stable state, and no further changes in swelling pressure occur during the subsequent process of suction reduction. This observation indicates that the bentonite reaches its hydration limit when the suction reaches a certain threshold, resulting in the end of the hydration process.

This phenomenon could be explained by a change in the matric suction of bentonite during hydration. For bentonite, the adsorption component (physico-chemical effects) is the dominant component of matric suction, which arises from the presence of a substantial quantity of active montmorillonite minerals. During wetting, as montmorillonite crystals gradually become hydrated, adsorption (physico-chemical effects) gradually decreases. Ideally, complete hydration of all montmorillonite crystals within the bentonite would lead to the complete elimination of adsorptive suction (physico-chemical effects). Nevertheless, the confined condition introduces a limitation on the hydration process in which certain montmorillonite crystals may not undergo complete hydration as the ingress of water molecules into the bentonite ceases. Consequently, residual adsorption may persist within the material [23]. In this regard, as the bentonite specimen reaches the saturation state there may be residual adsorptive suction, indicating that matric suction may not fully dissipate and reach zero. Afterwards, despite continued decrease in the applied externally controlled suction, the confined condition prohibits the further hydration of montmorillonite crystals. As a result, the residual adsorption in bentonite remains unaffected by the application of externally controlled suction, indicating that further reduction in residual adsorption is not achievable. Thus, the swelling pressure of the bentonite cannot change further.

**Figure 5:**
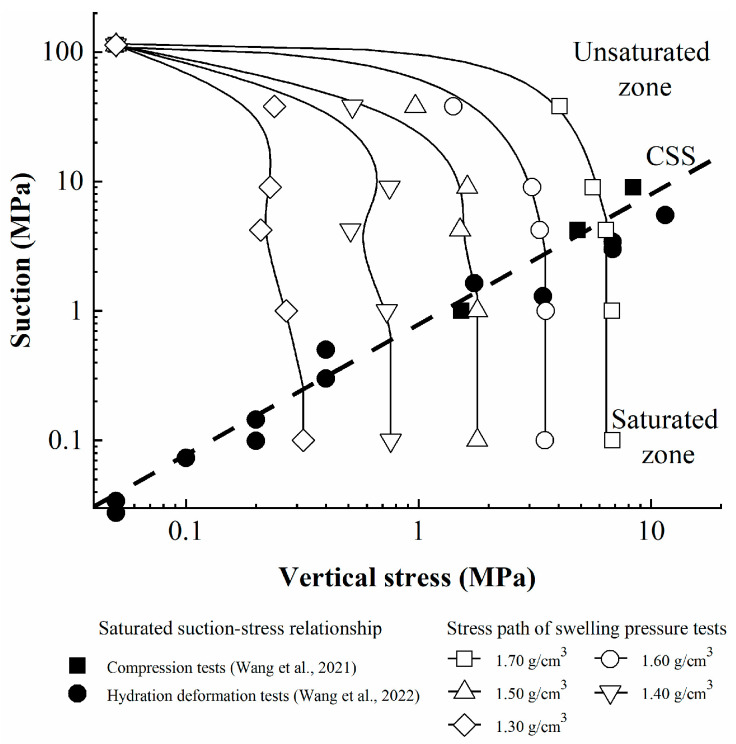
The stress path of the swelling pressure tests with suction control.

The suction threshold value upon hydration (suction decrease) can be defined as the saturated suction, which represents the residual adsorption suction resulting from the presence of unhydrated montmorillonite crystals. Wang et al. [23,24] found the existence of saturated suction during controlled suction compression tests and swelling deformation tests conducted on GMZ bentonite, the same material used in this study. The relationship between saturated suction and stress state has been analyzed when bentonite reach saturated state, as shown in Figure 5. The results show that a unique stress–suction relationship exists when bentonite reaches the saturation state, regardless of the stress path and dry density of specimen. Wang et al. [23] defined the curve as the critical saturated state (CSS) curve, which divides the s–p plane into unsaturated and saturated zones.

The swelling pressure–suction relationship obtained in this work, which represents the stress path of constant volume swelling pressure tests, is plotted in Figure 5. An obvious phenomenon can be seen in that the swelling pressure–suction curves seem to be divided into unsaturated and saturated segments. When the stress path reaches the CSS curve, the swelling pressure achieves stable state. Therefore, these results indicate that the CSS curve is unique in the stress space independently of the stress path.

In addition, for the specimen with lower dry density, the final swelling pressure when saturated is lower, and the corresponding saturated suction is lower. In case of the sample with lower dry density, there was more space for swelling; thus, montmorillonite crystals may have experienced relatively adequate hydration with weak adsorptive suction (physico-chemical effects), resulting in lower saturated suction.

**Figure 6:**
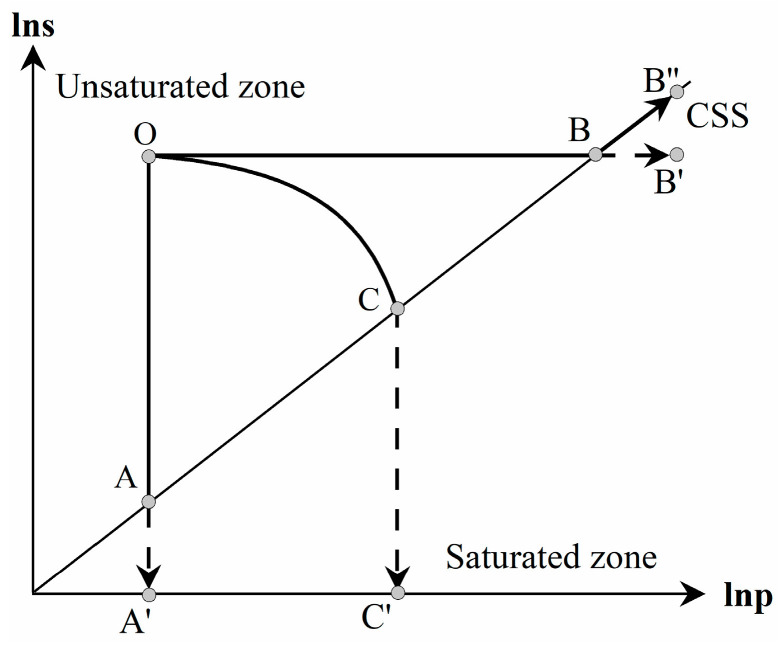
The three stress paths in the s–p space with the CSS curve.

As shown in Figure 6, the CSS curve in the s–p space represents the correlation between saturation suction (residual adsorption) and the current stress state for saturated bentonite. The saturation suction is the minimum suction achievable upon the applied load. Therefore, the stress state of bentonite would not fall under the CSS curve. The rule stress space of the unsaturated expansive soil is the zone between the s-axis and the CSS curve, which is different from the conventional stress space of unsaturated soil. The location of the CSS curve reflects the swelling capability (physico-chemical effect) of the expansive soil. The larger the mineral content of montmorillonite, the stronger the swelling capability of the soil, and the more the angle of the CSS curve deviates from the p-axis. In case of non-expansive soil, the CSS curve coincides with the p-axis, and the stress space degrades into the conventional one of unsaturated soil.

The three stress paths in the s–p space are plotted in Figure 6. When the stress state reaches the CSS curve, irrespective of the path it follows, the initially unsaturated specimen transitions to saturation. In the case of stress path OAA′ in Figure 6 for the hydration test under constant applied stress, the real stress path of bentonite is OA. In the case of stress path OBB′ in Figure 6 for the compression test under constant controlled suction, the actual stress path of bentonite follows OBB″. In the case of stress path OCC′ in Figure 6 for the constant volume swelling pressure test, the real stress path of bentonite is OC. 

Therefore, the analysis above shows that with the introduction of the CSS curve into the s–p space, the conventional constitutive framework of unsaturated soil based on independent variable approach can be expanded to effectively describe the mechanical characteristics of expansive soil.

## 5. Conclusions

In this study, constant volume swelling pressure tests with controlled suction were conducted on compacted GMZ bentonite. The swelling pressure–suction relationship and corresponding mechanism for specimens with different dry densities were analyzed during hydration. The following conclusions can be drawn.

The swelling pressure of specimens increased significantly with increasing dry density. A clear linear relation was observed between the final swelling pressure and the dry density of the specimen, as shown in the semilogarithmic plot.

The shape of the swelling pressure–suction curves during hydration depended on the dry density. For specimens with low dry densities, the swelling pressure evolution curve exhibited the characteristic of the swelling pressure initially increasing, then decreasing, and then increasing again. When the dry density was higher, the phenomenon became non-obvious.

The swelling pressure–suction curves were observed to be divided into unsaturated and saturated segments, proving the unique existence of the CSS curve in the stress space independent of the stress path. By introducing the CSS curve into the s–p space, the conventional stress space of unsaturated soil can be expanded to that of unsaturated expansive soil.

## Figures and Tables

**Table 1:** Basic properties of GMZ01 bentonite.

Property	Description
Specific gravity	2.66
Liquid limit (%)	276
Plastic limit	37
Cation-exchange capacity (meq/100 g)	77.3 (43.36% Na^+^, 29.14% Ca^2+^, 12.33% Mg^2+^, 2.51% K^+^)
Main minerals	75.4% Montmorillonite, 11.7% Quartz, 4.3% Feldspar, 7.3% Cristobalite

**Table 2:** Specifications of the swelling pressure tests.

Tests	Dry Density (g/cm^3^)	Suction Paths (MPa)
1	1.30	113–38–9–4.2–1–0
2	1.40	113–38–9–4.2–1–0
3	1.50	113–38–9–4.2–1–0
4	1.60	113–38–9–4.2–1–0
5	1.70	113–38–9–4.2–1–0

## Data Availability

All data, models, or code that support the findings of this study are available from the corresponding author.

## References

[1] Komine H., Ogata N. (1996). Prediction for swelling characteristics of compacted bentonite. Can. Geotech. J..

[2] Villar M.V. (2006). Infiltration tests on a granite/bentonite mixture: Influence of water salinity. Appl. Clay Sci..

[3] Wen Z.J. (2006). Physical property of China’s buffer material for high-level radioactive waste repositories. Chin. J. Rock Mech. Eng..

[4] Ye W.M., Chen Y.G., Chen B., Wang Q., Wang J. (2010). Advances on the knowledge of the buffer/backfill properties of heavily-compacted GMZ bentonite. Eng. Geol..

[5] Siddiqua S., Blatz J., Siemens G. (2011). Evaluation of the impact of pore fluid chemistry on the hydromechanical behavior of clay-based sealing materials. Can. Geotech. J..

[6] Chen T., Du M., Yao Q. (2021). Evolution of Hydraulic Conductivity of Unsaturated Compacted Na-Bentonite under Confined Condition-Including the Microstructure Effects. Materials.

[7] Wang Y., Teng J., Huang Q., Wang W., Ren Z. (2022). Insight on the Void Ratio–Suction Relationship of Compacted Bentonite during Hydration. Materials.

[8] Ye W.M., Wang Y., Wang Q., Chen Y.G., Chen B. (2020). Stress-dependent temperature effect on the swelling behavior of compacted GMZ bentonite. B. Eng. Geol. Environ..

[9] Ye W.M., Schanz T., Qian L.X., Wang J., Arifin (2007). Characteristics of swelling pressure of densely compacted Gaomiaozi bentonite GMZ01. Chin. J. Rock Mech. Eng..

[10] Wang Q., Tang A.M., Cui Y.J., Delage P., Gatmiri B. (2012). Experimental study on the swelling behaviour of bentonite/claystone mixture. Eng. Geol..

[11] Lloret A., Romero E., Villar M.V. (2004). FEBEX II Project Final Report on Thermo-Hydro-Mechanical Laboratory Tests.

[12] Tang A.M., Cui Y.J., Barnel N. (2008). Thermo-mechanical behaviour of a compacted swelling clay. Géotechnique.

[13] Bailie W., Tripathy S., Schanz T. (2010). Swelling pressures and one-dimensional compressibility behaviour of bentonite at large pressures. Appl. Clay Sci..

[14] Lloret A., Villar M.V., Sanchez M., Gens A., Pintado X., Alonso E.E. (2003). Mechanical behavior of heavily compacted bentonite under high suction changes. Géotechnique.

[15] Yigzaw Z.G., Cuisinier O., Massat L., Masrouri F. (2016). Role of different suction components on swelling behavior of compacted bentonites. Appl. Clay Sci..

[16] Romero E., Gens A., Lloret A. (2003). Suction effects on a compacted clay under non-isothermal conditions. Géotechnique.

[17] Agus S.S., Arifin Y.F., Tripathy S., Schanz T. (2013). Swelling pressure-suction relationship of heavily compacted bentonite-sand mixtures. Acta Geotech..

[18] Liu Y.M., Wen Z.J. (2003). An investigation of the physical properties of clayey materials used in nuclearwaste disposal at great depth. Miner. Rocks.

[19] Delage P., Howat M.D., Cui Y.J. (1998). The relationship between suction and swelling properties in a heavily compacted unsaturated clay. Eng. Geol..

[20] Tang A.M., Cui Y.J. (2005). Controlling suction by the vapour equilibrium technique at different temperatures and its application in determining the water retention properties of MX80 clay. Can. Geotech. J..

[21] Dixon D.A., Graham J., Gray M.N. (1999). Hydraulic conductivity of clays in confined tests under low hydraulic gradients. Can. Geotech. J..

[22] Villar M.V. (2002). Thermo-hydro-mechanical characterisation of a bentonite from Cabo de Gata. A Study Applied to the Use of Bentonite as Sealing Material in High Level Radioactive Waste Repositories.

[23] Wang Y., Ye W.M., Wang Q., Chen Y., Cui Y.J. (2022). A critical saturated state-based constitutive model for volumetric behavior of compacted bentonite. Can Geotech J..

[24] Wang Y., Ye W.M., Chen B., Chen Y.G., Cui Y.J. (2021). A nonlinear normal consolidation line for bentonite in e-logp space. Eng. Geol..

